# Prevalence of Multidrug-Resistant Tuberculosis and Its Association With Previous Treatment History in Adults

**DOI:** 10.7759/cureus.88204

**Published:** 2025-07-17

**Authors:** Muhammad Ahmad Mughal, Adnan Imran, Hashmat Ullah Khan, Muhammad Farooq, Amna Ikram, Fizza Arshad, Razwan Ashraf, Fahmida Khatoon

**Affiliations:** 1 Community Medicine, Wah Medical College, Wah, PAK; 2 Medicine, Institute Walsall Manor Hospital, Walsall, GBR; 3 Medicine, Lady Reading Hospital - Medical Teaching Institute (MTI), Peshawar, PAK; 4 Medicine, Watim Medical College, Rawalpindi, PAK; 5 Medicine, Basic Health Unit (BHU) Chandar Nagar, Nankana Sahib, PAK; 6 Medicine and Surgery, Saad Medical Complex Faisalabad, Faisalabad, PAK; 7 Medicine, Aziz Bhatti Shaheed Teaching Hospital, Gujrat, PAK; 8 Biochemistry, University of Ha’il, Ha’il, SAU

**Keywords:** diabetes mellitus, drug resistance, multidrug-resistant tuberculosis, previous treatment, smoking

## Abstract

Background

Multidrug-resistant tuberculosis (MDR-TB) poses a growing threat to global tuberculosis (TB) control efforts, particularly in high-burden countries like Pakistan. This study aimed to determine the prevalence of MDR-TB among adult pulmonary TB patients and evaluate its association with previous treatment history and other potential risk factors.

Methods

A cross-sectional analytical study was conducted at a tertiary care hospital in Lahore, Pakistan, from January to June 2024. A total of 250 adult patients with microbiologically confirmed pulmonary TB were enrolled using a non-probability consecutive sampling technique. Sociodemographic and clinical data were collected using a structured questionnaire. Sputum samples were tested using the GeneXpert MTB/RIF assay, and rifampicin-resistant samples were further analyzed by culture-based drug susceptibility testing to confirm MDR-TB. Data were analyzed using SPSS Version 26. Logistic regression was used to identify independent predictors of MDR-TB.

Results

The overall prevalence of MDR-TB was 18.8% (47/250). MDR-TB was significantly more prevalent in previously treated patients (40.0%) compared to newly diagnosed cases (6.9%) (p < 0.001). On multivariate analysis, previous TB treatment (adjusted odds ratio [AOR] = 7.85; 95% CI: 3.85-16.00), smoking history (AOR = 2.13; 95% CI: 1.02-4.45), and diabetes mellitus (AOR = 2.75; 95% CI: 1.33-5.68) were independently associated with MDR-TB. Age and gender were not significantly associated with MDR-TB.

Conclusion

The study revealed a high prevalence of MDR-TB, especially among previously treated patients. Previous TB treatment, smoking, and diabetes mellitus were key risk factors. These findings emphasize the importance of comprehensive drug resistance screening and the integration of non-communicable disease management and tobacco control into TB care strategies.

## Introduction

Tuberculosis (TB) remains one of the leading causes of mortality globally. In 2021, an estimated 10.6 million individuals were affected by TB, marking a 4.5% increase from 10.1 million in 2020. Globally, TB caused approximately 1.6 million deaths in 2021, emphasizing its high mortality burden. Pakistan is among the top five high-burden TB countries globally. According to the World Health Organization (WHO) Global TB Report 2023, an estimated 620,000 people developed TB in Pakistan in 2022, with a mortality rate of 44 deaths per 100,000 population, excluding HIV-positive individuals [1]. Despite the availability of national-level data, detailed regional surveillance data for cities such as Lahore remains limited and should be interpreted cautiously.

Despite intensified control efforts, drug-resistant tuberculosis (DR-TB) continues to pose a major public health challenge worldwide [2]. Among DR-TB forms, resistance to rifampicin (RIF) - the most potent first-line anti-TB drug - is most concerning. When resistance occurs to both RIF and isoniazid (INH), the condition is classified as multidrug-resistant TB (MDR-TB) [3]. Treatment of MDR-TB or rifampicin-resistant TB (RR-TB) requires second-line anti-TB agents, which are less effective, are more toxic, and require prolonged therapy. In 2021, an estimated 450,000 MDR/RR-TB cases were reported globally.

Pakistan is among the seven high-burden countries that together accounted for two-thirds of MDR/RR-TB cases globally in 2021. National estimates suggest that MDR/RR-TB was present in 3.6% of new TB cases and 18% of previously treated patients [1]. Pakistan faces a substantial burden of MDR-TB, with an estimated 11,000 cases reported in 2022. The rate of MDR-TB is notably higher among previously treated individuals in the country [4].

Patients are classified as previously treated if they have received anti-TB therapy for at least one month. These cases are further categorized based on their previous treatment outcome. Treatment failure is defined as a patient whose sputum smear or culture remains positive at month 5 or later during treatment [5,6] and has been identified as a significant risk factor for developing MDR-TB [7]. Emerging research has also highlighted the potential role of genetic mutations in *Mycobacterium tuberculosis* - particularly in the rpoB, katG, and inhA genes - as key contributors to drug resistance. These genetic alterations interfere with the drug targets or activation mechanisms and lead to therapeutic failure.

Various factors contribute to treatment failure and resistance development, including non-standard treatment regimens, suboptimal dosing schedules, and poor adherence [8,9]. First-line TB drugs such as RIF, INH, ethambutol, and pyrazinamide exhibit concentration-dependent activity. For optimal pharmacokinetics - particularly Cmax and Cmax/AUC - drugs should be administered once daily at the correct dose. Deviations, such as split-dose administration, may impair therapeutic efficacy [10]. Additionally, food-drug interactions, especially with RIF and INH, can reduce drug bioavailability, further risking treatment failure if taken incorrectly [11].

Poor adherence to DOTS (Directly Observed Treatment Short-course) strategies remains a major driver of resistance. DOTS, endorsed by WHO, ensures supervised administration of medication, often by trained family members or community workers, to enhance adherence [12]. In Pakistan, the National TB Control Program (NTP) adopts WHO-endorsed DOTS strategies, which are implemented primarily through basic health units (BHUs) and rural health centers (RHCs). Trained community health workers and family members supervise medication intake under program guidelines. However, regional variations in adherence, education, and drug availability affect treatment outcomes.

Two formulations are used for TB therapy in Pakistan: fixed-dose combinations (FDCs) and separate drugs. FDCs combine RIF, INH, pyrazinamide, and ethambutol into a single pill, simplifying administration and improving adherence [13]. The initial two months of intensive therapy involve 4-FDC (RIF, INH, pyrazinamide, and ethambutol) administration, followed by 2-FDC (INH and RIF) regimens during the four-month continuation phase [14]. These formulations are recommended by WHO and endorsed by the Ministries of Health in both countries [3]. However, inconsistencies in drug distribution and lack of coordination across health facilities may hinder uniform implementation.

Pakistan’s public healthcare system is structured into three levels: primary (BHUs and RHCs), secondary (tehsil and district headquarters hospitals), and tertiary (teaching and specialized hospitals). TB treatment is primarily initiated at the primary care level under the NTP, while complex or resistant cases are referred to tertiary care centers such as the one where this study was conducted.

Treatment failure contributes to ongoing transmission and increases the risk of MDR-TB emergence in the community [15]. Second-line therapy is costlier, less effective, associated with more side effects, and linked to higher mortality rates [16]. Preventing treatment failure in drug-sensitive TB is thus crucial to improving outcomes and curbing resistance development [17].

To strengthen TB control efforts and reduce MDR-TB risk, it is essential to understand how prior treatment and its quality influence resistance patterns. This study aimed to determine the prevalence of MDR-TB and assess its association with previous anti-TB treatment history in adult pulmonary TB patients at a tertiary care center in Lahore, Pakistan.

## Materials and methods

This study was conducted at the Department of Pulmonology, Jinnah Hospital Lahore, Lahore, Pakistan, a facility equipped with a dedicated TB diagnosis and treatment unit catering to both new and retreatment TB cases. The study was approved by the Institutional Review Board (IRB) of Lady Reading Hospital MTI Peshawar (361/LHR/MT, dated 02/01/2024). Written informed consent was obtained from all participants prior to their enrollment.

The study followed a cross-sectional analytical design and was conducted over a period of six months (January 2024 to June 2024). The target population included adult patients diagnosed with pulmonary TB and registered with the hospital’s TB treatment center during the study period.

The sample size was calculated using the OpenEpi online sample size calculator for cross-sectional studies. Assuming an expected prevalence of MDR-TB among previously treated patients to be 18%, with a confidence level of 95% and a margin of error of 5%, the required sample size was estimated to be 227. To accommodate potential dropouts and incomplete data, the sample size was increased to 250 participants.

A non-probability consecutive sampling technique was employed to recruit participants. All eligible patients who presented at the TB clinic during the study period and met the inclusion criteria were invited to participate until the desired sample size was reached.

Inclusion criteria comprised adult patients aged 18 years and above, of either gender, who had a microbiologically confirmed diagnosis of pulmonary TB through GeneXpert MTB/RIF assay or sputum culture, and who were either newly diagnosed or had a documented history of previous anti-TB treatment. Exclusion criteria included patients with extrapulmonary TB, those co-infected with HIV, patients with incomplete clinical records, and individuals who refused to give informed consent.

A structured questionnaire was used to collect sociodemographic and clinical data, including age, sex, residence, occupation, smoking status, comorbidities, and history of previous TB treatment. The treatment history was verified through patient-held records and NTP databases, including the nature, duration, and completion status of previous therapy.

All patients underwent sputum sample collection in sterile containers following standard biosafety protocols. Each sample was subjected to GeneXpert MTB/RIF testing to confirm the presence of *Mycobacterium tuberculosis* and to detect RIF resistance. Samples found resistant to RIF on GeneXpert were subjected to further culture and drug susceptibility testing (DST) using the BACTEC MGIT 960 system to determine resistance to both RIF and INH, confirming multidrug resistance as per WHO criteria. All diagnostic tests were performed at the hospital's biosafety level 3 (BSL-3) laboratory, accredited by the National TB Reference Laboratory.

Participants were then stratified into two groups: treatment-naïve (newly diagnosed) and previously treated (including relapse, default, and treatment failure). The prevalence of MDR-TB was calculated for each group. The primary outcome variable was the presence of MDR-TB, while the primary independent variable was the history of previous TB treatment. Other covariates included age, gender, smoking, and comorbidities (e.g., diabetes mellitus).

Data were entered and analyzed using Statistical Package for the Social Sciences (SPSS) Version 26.0 (IBM Corp., Armonk, NY). Descriptive statistics were used to summarize categorical variables as frequencies and percentages and continuous variables as means and standard deviations. The prevalence of MDR-TB was calculated overall and within subgroups. The association between MDR-TB and previous treatment history was assessed using the chi-square test. Binary logistic regression analysis was performed to identify predictors of MDR-TB, adjusting for potential confounders. Odds ratios (ORs) with 95% confidence intervals (CIs) were reported, and a p-value of less than 0.05 was considered statistically significant.

All laboratory procedures adhered strictly to biosafety guidelines and followed standard operating procedures under supervision of the hospital’s microbiology department. The confidentiality and privacy of participants were maintained throughout the study.

## Results

A total of 250 adult patients with confirmed pulmonary TB were enrolled. Of these, 160 (64.0%) were newly diagnosed cases without prior anti-TB treatment, and 90 (36.0%) had received treatment previously. The mean age was 41.7 ± 13.2 years, with 144 (57.6%) males and 106 (42.4%) females. MDR-TB was found in 47 (18.8%) participants, with a disproportionately high frequency among previously treated patients.

Table 1 summarizes the demographic and baseline clinical features of the participants. The majority of patients were in the age group of 31-50 years, with a slightly higher proportion of males. A considerable number of participants had a history of smoking (29.6%) and diabetes mellitus (27.2%). Previous TB treatment was documented in over one-third of the study population.

**Table 1:** Sociodemographic and clinical characteristics of study participants (N = 250)

Variable	n (%)
Age group (years)	
18–30	62 (24.8%)
31–50	109 (43.6%)
>50	79 (31.6%)
Gender	
Male	144 (57.6%)
Female	106 (42.4%)
Smoking status	
Smoker/ex-smoker	74 (29.6%)
Non-smoker	176 (70.4%)
Comorbidities	
Diabetes mellitus	68 (27.2%)
Hypertension	32 (12.8%)
None	150 (60.0%)
Previous tuberculosis treatment	
Yes	90 (36.0%)
No	160 (64.0%)

Table 2 demonstrates a highly significant association between prior TB treatment and the presence of MDR-TB (χ² = 43.67, p < 0.001). Among patients who had received previous treatment, 40.0% had MDR-TB compared to only 6.9% in newly diagnosed individuals.

**Table 2:** Prevalence of MDR-TB according to treatment history (N = 250) Significance level: p < 0.05 considered statistically significant. *Indicates a significant p-value. MDR-TB, multidrug-resistant tuberculosis

Treatment History	Total, n (%)	MDR-TB, n (%)	Prevalence (%)
Newly diagnosed	160 (64.0%)	11 (6.9%)	6.9%
Previously treated	90 (36.0%)	36 (40.0%)	40.0%
Total	250 (100%)	47 (18.8%)	18.8%
χ² (chi-square)	-	-	43.67
p-value	-	-	<0.001*

Table 3 explores univariate associations between MDR-TB and various study variables. Statistically significant associations were observed with previous TB treatment (p < 0.001), smoking (p = 0.013), and diabetes mellitus (p = 0.002). Gender and age group were not significantly associated with MDR-TB.

**Table 3:** Univariate association between MDR-TB and study variables (N = 250) Significance level: p < 0.05 considered statistically significant. *Indicates a significant p-value. MDR-TB, multidrug-resistant tuberculosis

Variable	MDR-TB, n (%)	Non-MDR-TB, n (%)	χ² Value	p-Value
Gender				
Male	30 (63.8%)	114 (56.2%)	0.60	0.439
Female	17 (36.2%)	89 (43.8%)		
Age group (years)				
18–30	7 (14.9%)	55 (27.1%)	3.22	0.200
31–50	20 (42.6%)	89 (43.8%)		
>50	20 (42.6%)	59 (29.1%)		
Smoking status				
Smoker/ex-smoker	21 (44.7%)	53 (26.1%)	6.14	0.013*
Non-smoker	26 (55.3%)	150 (73.9%)		
Diabetes mellitus				
Yes	22 (46.8%)	46 (22.7%)	9.83	0.002*
No	25 (53.2%)	157 (77.3%)		
Previous treatment				
Yes	36 (76.6%)	54 (26.6%)	43.67	<0.001*
No	11 (23.4%)	149 (73.4%)		

Table 4 presents the results of multivariate logistic regression. After adjusting for confounding variables, previous TB treatment remained the most significant predictor of MDR-TB (adjusted odds ratio [AOR]: 7.85; p < 0.001). Smoking (AOR: 2.13; p = 0.043) and diabetes mellitus (AOR: 2.75; p = 0.006) also showed statistically significant independent associations. Gender and older age were not significant predictors.

**Table 4:** Multivariate logistic regression analysis for predictors of MDR-TB (N = 250) Significance level: p < 0.05 considered statistically significant. *Indicates a significant p-value. MDR-TB, multidrug-resistant tuberculosis; TB, tuberculosis

Predictor Variable	Adjusted Odds Ratio	95% Confidence Interval	p-Value
Previous TB treatment	7.85	3.85–16.00	<0.001*
Smoking history	2.13	1.02–4.45	0.043*
Diabetes mellitus	2.75	1.33–5.68	0.006*
Age > 50 years	1.34	0.64–2.80	0.431
Male gender	0.96	0.49–1.88	0.910

Out of 250 TB patients, 47 (18.8%) had MDR-TB. A strong and statistically significant association was observed between MDR-TB and previous treatment history, with MDR-TB seen in 36 (40.0%) of previously treated patients versus 11 (6.9%) of newly diagnosed cases. After controlling for confounders, previous TB treatment, smoking, and diabetes mellitus emerged as significant independent predictors of MDR-TB. No significant association was found with gender or age after adjustment.

These findings emphasize the urgent need for focused screening, early resistance testing, and tailored interventions for individuals with a history of TB treatment, smokers, and diabetic patients to limit the spread and burden of MDR-TB.

## Discussion

This study examined the prevalence of MDR-TB and its association with previous treatment history and selected risk factors in adult patients at a tertiary care hospital. The overall prevalence of MDR-TB was found to be 18.8%, with a markedly higher burden in patients who had a history of previous TB treatment (40.0%) compared to those newly diagnosed (6.9%). These findings highlight the critical need to address retreatment cases more aggressively in national TB control programs.

The association between a history of prior TB treatment - particularly among patients with incomplete or failed treatment - and MDR-TB observed in this study (AOR = 7.85; p < 0.001) underscores the importance of treatment quality and adherence rather than the treatment itself. Several studies have reported similar findings. For instance, a study from Pakistan reported a 42% prevalence of MDR-TB among previously treated patients, comparable to our 40% rate [18]. That study was also conducted at a tertiary care facility, which, similar to our setting, typically receives complicated or drug-resistant cases referred from peripheral centers. This elevated risk is likely attributable to factors such as poor adherence to drug regimens, substandard prescription practices, and interruptions in treatment courses, all of which can lead to acquired resistance [19]. Furthermore, the use of suboptimal regimens and inappropriate drug combinations in private-sector treatment settings has also been implicated in the development of resistance [20,21].

The observed 6.9% prevalence of MDR-TB among newly diagnosed patients, while significantly lower than in previously treated individuals, still exceeds WHO's estimated 4.1% global average [22]. Other studies have reported similar trends; for example, an Indian study noted a prevalence of 10.4% among new cases [23], and research in Ethiopia reported rates ranging from 6% to 11% in treatment-naïve patients [24]. The presence of MDR-TB in new patients suggests ongoing community transmission of resistant strains and highlights the need for universal DST, even in patients with no history of anti-TB therapy.

Smoking was found to be significantly associated with MDR-TB in our cohort (AOR = 2.13; p = 0.043). This is in line with several studies that identify tobacco use as a major risk factor for TB progression and resistance [25-27]. Smoking has been shown to impair pulmonary immune defenses and alter the pharmacokinetics of anti-TB drugs, potentially delaying sputum conversion and increasing the risk of treatment failure and resistance. Moreover, smokers may have poorer treatment adherence, which is a known driver of resistance development [28].

The prevalence of diabetes mellitus was significantly higher among MDR-TB patients (46.8%) compared to non-MDR-TB patients (22.7%), and diabetes was an independent predictor of MDR-TB (AOR = 2.75; p = 0.006). This finding is consistent with several studies reporting that diabetic patients are at an increased risk of developing DR-TB due to impaired immune response, altered drug metabolism, and higher rates of treatment failure [29,30]. Additionally, diabetes-associated complications such as gastroparesis may affect the absorption of TB medications, further contributing to subtherapeutic drug levels and resistance [31].

Although the age group of >50 years accounted for a large proportion of MDR-TB cases (42.6%), age was not a statistically significant independent predictor in the multivariate model (AOR = 1.34; p = 0.431). Similar non-significant associations have been reported in other regional studies [32]. However, some reports from Europe and China have noted higher MDR-TB risk in middle-aged populations (35-44 years) [22,33]. The inconsistency may reflect geographical differences in treatment practices and health system access.

Gender was also not significantly associated with MDR-TB in this study (AOR = 0.96; p = 0.910) despite a higher absolute number of MDR-TB cases in males (63.8%). This finding contrasts with reports from Egypt, Ghana, and Ethiopia, where males constituted up to 70% of MDR-TB cases [34-37]. The absence of statistical significance in our cohort may be due to the relatively balanced male-to-female ratio and suggests that gender alone may not be a strong independent predictor when adjusted for other risk factors.

The overall MDR-TB prevalence of 18.8% in our study is higher than global pooled estimates, which report MDR-TB prevalence at approximately 11.6% across all TB types [38]. However, our findings are consistent with data from high-burden countries in South Asia and parts of East Africa, where MDR-TB prevalence among previously treated patients can exceed 20% [38,39]. These patterns reflect challenges related to treatment adherence, weak regulation of drug prescription, and inadequate implementation of DOTS strategies in both public and private healthcare sectors.

This study was conducted in a single tertiary care hospital, which may limit the generalizability of the findings to broader populations. Resistance testing beyond RIF was performed only for GeneXpert-positive cases, potentially underestimating overall MDR-TB prevalence. Additionally, self-reported data on smoking and treatment history may be prone to recall bias.

The high burden of MDR-TB among previously treated patients, combined with the significant role of modifiable risk factors such as smoking and diabetes, underscores the need for targeted interventions. Strengthening routine DST, ensuring an uninterrupted supply of quality-assured TB medications, and integrating non-communicable disease management (such as diabetes care) into TB programs are essential steps. Public health strategies should also focus on tobacco cessation, enhanced patient counseling, and stricter implementation of directly observed therapy (DOT) to prevent treatment failure and the development of resistance. This study did not perform a subgroup analysis of previously treated TB patients (e.g., treatment failure, relapse, defaulters), which may have provided a more nuanced understanding of MDR-TB risk within this population. Additionally, data on pre-existing chronic lung disease and the interval between prior treatment completion and current diagnosis were not captured, limiting the exploration of these potentially significant associations.

## Conclusions

This study demonstrates a high prevalence of MDR-TB, particularly among previously treated adult patients. Prior TB treatment history was the strongest independent predictor of MDR-TB, followed by smoking and diabetes mellitus. The findings underscore the urgent need for comprehensive drug resistance screening, especially among retreatment cases, and highlight the importance of integrating tobacco cessation and diabetes management into TB control programs. Addressing these modifiable risk factors through targeted public health strategies and improving treatment adherence could significantly reduce the burden of MDR-TB in high-risk populations. Future multicenter and longitudinal studies are recommended to further explore the dynamics of resistance development and evaluate the impact of integrated interventions.
